# Can an ethics officer role reduce delays in research ethics approval? A mixed-method evaluation of an improvement project

**DOI:** 10.1136/bmjopen-2016-011973

**Published:** 2016-08-31

**Authors:** Mary Dixon-Woods, Chris Foy, Charlotte Hayden, Rustam Al-Shahi Salman, Stephen Tebbutt, Sara Schroter

**Affiliations:** 1 Institute of Public Health, Forvie Site, University of Cambridge School of Clinical Medicine, Cambridge, UK; 2 R&D, Gloucestershire Hospitals NHS Foundation Trust, Gloucester, UK; 3 North Bristol NHS Trust, Southmead Hospital, Bristol, UK; 4 Centre for Clinical Brain Sciences, School of Clinical Sciences, College of Medicine and Veterinary Medicine, University of Edinburgh, Edinburgh, UK; 5 Health Research Authority, London, UK; 6 BMJ, BMA House, London, UK

**Keywords:** research ethics, controlled study, process evaluation, quality improvement

## Abstract

**Objective:**

Frustration continues to be directed at delays in gaining approvals for undertaking health research in the UK. We aimed to evaluate the impact of an ethics officer intervention on rates of favourable opinions (approval) and provisional opinions (requiring revision and resubmission) and on the time taken to reach a final opinion by research ethics committees (RECs), to characterise how the role operated in practice, and to investigate applicants' views.

**Design:**

Mixed-method study involving (i) a 2-group, non-randomised before-and-after intervention study of RECs assigned an ethics officer and a matched comparator group; (ii) a process evaluation involving a survey of applicants and documentary analysis.

**Participants:**

6 RECs and 3 associated ethics officers; 18 comparator RECs; REC applicants.

**Results:**

Rates of provisional and favourable opinions between ethics officer and comparator RECs did not show a statistically significant effect of the intervention (logistic regression, p=0.26 for favourable opinions and p=0.31 for provisional opinions). Mean time to reach a decision showed a non-significant reduction (ANOVA, p=0.22) from 33.3 to 32.0 days in the ethics officer RECs compared with the comparator RECs (32.6 to 32.9 days). The survey (30% response rate) indicated applicant satisfaction and also suggested that ethics officer support might be more useful before submission. Ethics officers were successful in identifying many issues with applications, but the intervention did not function exactly as designed: in 31% of applicants, no contact between the applicants and the ethics officer took place before REC review.

**Limitations:**

This study was a non-randomised comparison cohort study. Some data were missing.

**Conclusions:**

An ethics officer intervention, as designed and implemented in this study, did not increase the proportion of applications to RECs that were approved on first review and did not reduce the time to a committee decision.

**Strengths and limitations of this study**

- This study is one of the few controlled evaluations of a quality improvement intervention in research ethics review.
- The study provides evidence that provision of an ethics officer role, at least as deployed in this project, does not shorten time to final decision by research ethics committees nor increase the proportion of applications that got a favourable opinion first time.
- Use of a process evaluation involving quantitative and qualitative methods enabled insights into the null effect of the intervention: it did not operate precisely as intended, and changes in culture and behaviour on the part of both research ethics committees and applicants are likely to be needed to secure change.
- The study was limited by missing data for the controlled evaluation and the process evaluation.
- The study would have been improved by further qualitative work, including interviews with participants and ethnography of meetings.

## Background

The importance and public benefits of health research have been repeatedly emphasised; research is now recognised as the cornerstone of evidence-based healthcare and critical for health system strengthening worldwide.^1–5^ However, the commitment to research is balanced against a number of other goals, including those relating to the control of risks and the safeguarding of research participants.^6^ How health research can best be governed and regulated remains an elusive question.^7^ Globally, reports of resentment and criticism continue to be directed at many parts of the system of ethical review and governance approvals.^8–10^ ^11–13^ How ethics and governance processes might be improved has remained, perhaps ironically, rarely formally evaluated.^14^ While the apparent arbitrary nature of decision-making is a frequent source of problem,^15^ ^16^ one potentially more easily tractable target for improvement is the delay associated with obtaining approvals for conducting research.^17^ In this article, we report a controlled study of an intervention aimed at reducing delays associated with research ethics committee (REC) approval.

**Figure 1:**
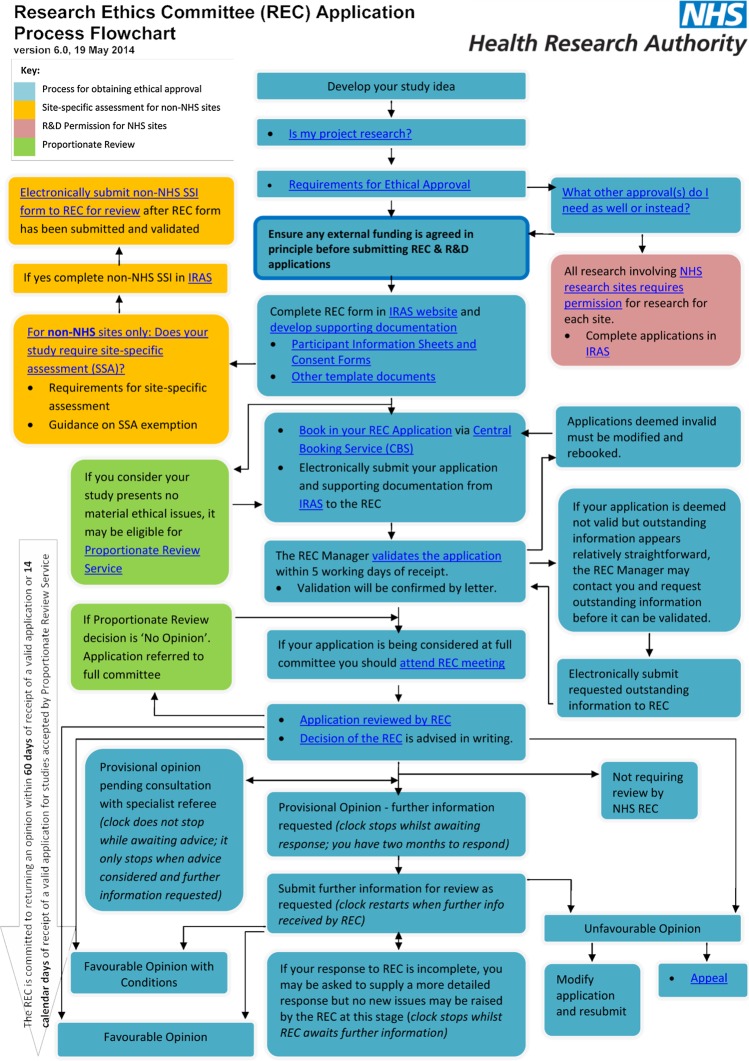
Overview of research ethics review process. CBS, Central Booking Service; IRAS, Integrated Research Application System; NHS, National Health Service; REC, research ethics committee; R&D, research and development; SSA, site-specific assessment; SSI, site-specific information.

Our study was based in the UK, where the research ethics system (figure 1) established under the Research Governance Framework^6^ requires that REC opinions be rendered within 60 days of submission. This target is consistently met.^18^ The Health Research Authority (HRA), which administers the system of RECs, has additionally set a stretch target that 95% of applications to full REC meetings have a final decision within 40 days. This target was met for 91% of applications in December 2014.^18^ However, these times to approval do not count periods while the ‘clock’ is stopped while applicants undertake any revisions that are required by RECs, meaning that in practice applicants may experience longer actual times to approval than those measured by formal clock times.

The length of time an application is in the process is strongly linked to the decision made when it is reviewed for the first time by an REC. At this first review, RECs have a choice of four decisions, known formally as *opinions*, which are recorded on an HRA electronic management information system. *Unfavourable opinions*, which remain rare (5% of applications to full committees, January–December 2015), result in applications being rejected altogether. *Favourable opinions,* which may be issued with a requirement for minor amendments known as ‘additional conditions’ (19% of 2015 applications) or without conditions (5%), allow applicants to proceed with their study without delay, since such applications do not need to be re-reviewed by the REC. A *provisional opinion* (71% of 2015 applications to full REC meetings), on the other hand, means that applicants must revise and resubmit their application and have it reviewed again by the REC, usually through subcommittee or chair's action. Such an opinion stops the clock on the 60-day limit while the applicants undertake revisions; the clock does not start again until they resubmit, potentially resulting in considerable time elapsing before a favourable opinion is obtained. Provisional opinions therefore have the potential to introduce delay, administrative burden and cost. They have remained the most common outcome of an application for REC approval. Yet, virtually all (99.9% in 2015) applications with a *provisional opinion* are given a *favourable opinion* once the application has been revised, suggesting that the issues detected at first review are almost always remediable.

Many of the frustrations associated with ethics review, by applicants and RECs, might be reduced if it were possible to get applications ‘right first time’ resulting in a favourable opinion. The issues that are likely to be raised by RECs at review are now well understood: they include ethical and procedural issues.^19–22^ At least some of the issues that contribute to provisional and unfavourable opinions are, in principle, preventable by better preparation on the part of applicants.^19–21^ One study, for example, found that 87% of applications not approved at first review had issues that included procedural violations, missing information, slip-ups such as errors in grammar and spelling, and discrepancies between different parts of the application.^20^

We hypothesised that an intervention to address these problems, involving more upfront effort invested in identifying issues with applications, could result in a higher rate of favourable opinions at first review and a consequent reduction in provisional opinions. We report a mixed-method, controlled evaluation of an ethics officer intervention designed to identify issues early, discuss them with applicants and communicate them to RECs. We aimed to assess whether assigning an ethics officer to an REC could increase the rate of favourable opinions and reduce the rate of provisional opinions and the time taken by the REC to reach a final opinion, as well as to characterise how the ethics officer role operated in practice and to investigate applicants' views of the role.

## Methods

A two-group, non-randomised, before-and-after intervention study was designed to compare the outcomes of applications submitted to RECs to which an ethics officer was assigned (the intervention group, referred to as ‘ethics officer RECs’) with matched control RECs without ethics officers (referred to as ‘comparator RECs’), together with a qualitative process evaluation. The intervention study was led by the HRA, guided by an advisory group at the design stage. The process evaluation was conducted and funded separately (see statements).

The project was deemed to be service evaluation and thus did not require review by an REC. All applicants to RECs in the ethics officer group were advised of the study and given the opportunity to opt-out of having their application considered by the ethics officer.

### The ethics officer intervention

An ethics officer was chosen as an intervention as other possible tactics to improve timeliness of review, including encouragement to RECs to use the ‘favourable with conditions’ option where appropriate, training for REC members and applicants and use of an electronic application handling system, had already been implemented. It was considered that provision of additional advice and support to applicants and committees might be a useful innovation. Accordingly, the ethics officer's role was to:

- Assess each application before the REC review meeting to identify any issues with the application that might prevent the REC from issuing a favourable opinion*.* Ethics officers were asked to use a brief eight-section review form (see box 1) to record all issues in applications at pre-review and during the meeting.
- Contact applicants to discuss the issues they had identified, including those that, in the opinion of the ethics officer, might result in changes being required by the REC. The ethics officer could discuss what changes might be made with a view to ensuring that the applicant would be better prepared to answer queries from the REC. The ethics officer could agree on clarifications and appropriate responses with applicants in advance of the REC meeting. Ethics officers were additionally requested to discuss whether applicants should attend in person or by phone. Ethics officers were asked to record the outcomes of these discussions with applicants on the review form.
- Report to the REC, on request, any issues noted on the pre-review form that the REC had not itself identified.

Box 1 **Sections on ethics officer review form**

1. Issues which may cause an application to not receive a favourable opinion (to be completed by the ethics officer in advance of the research ethics committee (REC) meeting and updated with any comments subsequent to the meeting).
2. Guidance or legal requirements for the REC to consider.
3. Prior assessment of application, including necessity for REC review, suitability of insurance arrangements, appropriate completion of study type on application, etc.
4. Advice received by the researcher.
5. Review of the participant information sheet and consent form (eg, to identify areas where they were inconsistent, incorrect or required additions and clarifications).
6. Attendance by the researcher (with ethics officers advised not to inform researchers that attendance was not required).
7. Time spent on application by the ethics officer.
8. Any other comments.

RECs were asked to conduct their review as normal. Neither applicants nor ethics officers were authorised to make any changes to the application or to submit additional documentation before the application was considered by the REC. This was because the systems in place validated applications on submission an electronic application handling system; applications could not then be amended until they had been reviewed by an REC unless they were formally withdrawn, resubmitted and validated again, thus potentially jeopardising dates booked for REC review when the application was initially submitted and assigned to a committee for a booked slot. It was considered that implementing a systems change to allow such resubmissions was outside the scope of this project. Ethics officers could, however, present a list of amendments (proposed solutions to problems) that they had agreed with the applicants before the applicant joined the meeting (or, if the applicant was not present, before the REC made its decision). Applicants could also refer to these agreed amendments during their meeting with the REC.

The ethics officer intervention sought to (i) decrease the proportion of *provisional opinions* (requiring resubmission and review) and (ii) increase the proportion of *favourable opinions with or without additional conditions,* which together we call ‘favourable opinions’.

The theory of change^23^ was that the ethics officer would reassure REC members that the revisions needed to comply with their expectations were understood by the applicants and would be implemented correctly, so that, rather than issuing a provisional opinion that would stop the clock and result in a resubmission and re-review, the REC would feel more confident about issuing a favourable opinion with additional conditions, and avoid unnecessary delay and the burden of the further approval route for both parties.

### REC selection and matching

In line with the HRA's wish to reduce the proportion of provisional opinions, the number of ethics officer and comparator RECs was chosen to detect, with 90% power and 5% significance level, a reduction in the proportion of *provisional* opinions from 70% to 50% in the ethics officer RECs.

Six RECs in England were chosen to have an ethics officer assigned to them for the 2013 calendar year, and thus formed the intervention group. These six RECs were located in centres which were considered ‘stable’, in that they were not then undergoing organisational change and had meeting dates that were compatible with the ethics officers' availabilities. Three ethics officers were appointed through an application and interview process following advertisement to all REC members and members of the National Research Ethics Advisors' Panel, with the aim of identifying individuals with appropriate expertise and experience. Each ethics officer was assigned a case load of two RECs.

The period January–April 2013 was used as a familiarisation period, and then the intervention was implemented during meetings held from May to November 2013 (the ‘intervention’ period). The same period a year earlier, including meetings held from May to November 2012, was used to provide preintervention baseline data.

We sought to match three comparator RECs to each of the six ethics officer RECs, so that 18 matched controls would be available. A panel of candidate RECs in England and Wales was created, with the comparator RECs matched in the first instance based on whether they were legally ‘recognised’ for review of Clinical Trials of Investigational Medicinal Products (CTIMPs). Of the six ethics officer RECs, one was recognised for review of CTIMPs including phase I trials in healthy volunteers; four were recognised for review of CTIMPs other than for healthy volunteers; and one was not recognised to review CTIMPs. Thus, five of the six ethics officer RECs were recognised to review CTIMPs (although each was ‘flagged’ for other types of review, such as for applications involving human tissue, qualitative methods, children, etc, which was not taken into account) and were matched with equivalent comparator RECs.

The next step in the matching process was to try to allow for the type of application the RECs were actually reviewing rather than what they were flagged to review. Each application to an REC is categorised as interventional or non-interventional, where ‘interventional’ is defined as a study where any intervention in care is evaluated, regardless of whether the intervention is medicinal. For each of the six ethics officer RECs, the three chosen comparators were those that had the same CTIMP recognition and achieved the closest match in the percentage of interventional studies reviewed from May to November 2012. Thus, there was no random element in the selection of comparators. It had been intended to match further by the percentage of provisional opinions issued by the RECs in the baseline ‘before’ period May–November 2012. However, this proved too difficult, mainly because one of the ethics officer RECs had a notably low provisional opinion rate (25%) during 2012, for which no match could be found.

### Quantitative data collection, outcomes and analysis

The primary outcomes were as follows:

- The number of provisional opinions at first review, expressed as a percentage of all applications reviewed.
- The number of favourable opinions (defined as favourable opinions at first review with or without additional conditions), expressed as a percentage of all applications reviewed.

The secondary outcome was the ‘clock time’ (the number of days taken by the REC from receipt of a valid application to issuing a final opinion, excluding time taken by the applicant to reply fully to queries).

To obtain relevant data, the HRA administrative database (Research Ethics Database (RED), now replaced by the HRA Assessment Review Portal (HARP)) that records all opinions made about applications and holds copies of the correspondence between applicants and REC was queried. The initial opinion on each application rendered by all RECs in the study over the baseline period May–November 2012 and the intervention period May–November 2013 was identified, and data on time to final opinion were extracted.

Statistical analysis was carried out using logistic regression of the two binary outcomes separately (favourable and provisional opinions), and by univariate analysis of variance for clock times. SPSS software (V.21) was used.

The logistic regressions treated each application to an REC as the unit of analysis. For the first of these, an approval (favourable or favourable with conditions) at first review was taken as the outcome of interest and was compared with all other outcomes. For the second regression, a provisional opinion at first review was compared with all other outcomes.

Explanatory variables entered into the regressions were as follows: year (2012 or 2013), ethics officer versus comparator REC and the interaction of these two terms. The first term was to uncover any trend in overall outcomes between 2012 and 2013. The second was to allow for baseline differences between ethics officer and comparator RECs, acknowledging that group allocation had not been at random. The third (interaction) term was the one that would show any effect of the ethics officer intervention. The remaining terms were CTIMP status, the percentage of interventional studies handled by the REC and the REC workload (measured as studies reviewed per meeting). The amount of administrative support available to RECs was approximately the same, so it was not included in the models.

As all of the explanatory variables were of importance to the model, the forced-entry method was used in preference to stepwise selection. Results are expressed as ORs with 95% CIs, together with the corresponding p values.

### Process evaluation of the applications to ethics officer RECs

A process evaluation was conducted involving a survey of applicants and an analysis of documents.

*Survey.* Applicants in the ethics officer intervention RECs were invited to complete an online survey feedback form (developed with input from a working group, but not piloted) after their application had completed review. Simple descriptive statistics were used to summarise the findings, and responses to open-ended questions were analysed thematically,^19^ facilitated by NVivo10.

*Documentary analysis.* REC opinion letters and the minutes of REC meetings relating to applications submitted in the baseline and intervention periods were obtained from the HRA database. These, together with the review forms completed by the ethics officers for the applications they handled, formed the ‘documentary data’ for each application. Box 1 shows the sections included in the ethics officer review form.

The documentary data were coded using a framework previously developed for categorising ‘issues’ in ethics applications,^19^ ^20^ ^24^ defined as any concern or query raised during ethical review. For purposes of this analysis, issues were organised into three categories: document issues, ethical issues and study issues (box 2). Requests by RECs for revisions to applications were coded as relating to documents, procedures or further information.

Box 2 **Categories of issues that might be identified in applications for ethical approval**

*Document issues* comprise any errors or inaccuracies with the consent forms for participants, the participant information sheets and similar documents relating to consent. Other document issues include those relating to general practitioner letters, errors in the electronic application form and associated documentation (eg, investigator CVs and consent forms) and inconsistencies in various parts of the application or absence of required documents and wording.

*Ethical issues* include those related to the care and safety of research participants, confidentiality and informed consent.

*Study issues* refer to those concerning the study design and methods, justification of risk over the intended benefit, inclusion and exclusion criteria, along with the appropriateness of recruitment methods.

Initial coding, facilitated by NVivo10 qualitative analysis software, was conducted by one researcher (CH). As a check on consistency of coding, a random sample of 10 (9%) preintervention documentary data sets and 12 (12%) during-intervention documentary datasets were also blind-coded by an independent coder (slightly more during-intervention documents were blind-coded to check consistency of the review form analysis). No significant discrepancies were noted between preintervention and intervention coding. Some minor inconsistencies were noted in interpretation of the coding framework; these were resolved by discussion.

## Results

The six ethics officer RECs were collectively assigned 171 applications during the baseline period (May–November 2012) and 192 applications during the intervention period (May–November 2013). The 18 comparator RECs were assigned 528 applications during the baseline period and 551 during the intervention period. Ethics officer RECs considered a mean 4.99 applications per meeting, while comparator RECs considered a mean 5.03 applications.

Exact matching by CTIMP review status was achieved, but the percentage of interventional studies actually reviewed could not be matched in all cases. One of the ethics officer RECs reviewed an unusually high percentage of interventional studies (62%) in the 2012 baseline period, for which no match could be found. One of the ethics officer RECs and two of its comparators reviewed no interventional studies from May to November 2012, despite all being recognised for CTIMPs.

### Proportion of favourable and provisional opinions

*Favourable opinions:* Ethics officer RECs were slightly more likely than comparator RECs to give favourable opinions both before (33% vs 29%) and during (33% vs 26%) the intervention period (table 1). During the intervention period, the percentage of favourable opinions in the ethics officer RECs remained the same, while there was a small decrease in the comparator RECs.

**Table 1:** Number and percentage of favourable and provisional opinions

	May–November 2012 (baseline)	May–November 2013 (intervention period)	Total
Favourable opinions			
Ethics officer REC	56/171 (32.7)	63/192 (32.8)	119/363 (32.8)
Comparator REC	155/528 (29.4)	133/551 (26.2)	288/1079 (26.7)
Total	211/699 (30.1)	196/743 (26.4)	407/1442 (28.2)
Provisional opinions			
Ethics officer REC	98/171 (57.3)	110/192 (57.3)	208/363 (57.3)
Comparator REC	334/528 (63.3)	378/551 (68.6)	712/1079 (66.0)
Total	432/699 (61.8)	478/743 (65.7)	920/1442 (63.8)

REC, research ethics committee.

*Provisional opinions:* Ethics officer RECs issued a smaller percentage of provisional opinions, both before and during the intervention, than did the comparator RECs. In the ethics officer RECs, the percentage of provisional opinions stayed constant (57.3%) throughout the study period. In the comparator group, provisional opinions increased by 5% between 2012 and 2013.

Analysis showed that the variable dominating the opinion outcome was whether or not the REC was recognised to review CTIMPs (table 2). A CTIMP-recognised REC was much less likely to issue a favourable opinion and much more likely to issue a provisional opinion. A modest but statistically significant effect of year was also found: more provisional and fewer favourable opinions were issued in 2013 compared with 2012 (table 2). However, the difference on this variable between ethics officer and comparator RECs was not statistically significant in either analysis. There were also no significant effects due to the baseline percentage of interventional studies, nor of REC workload (studies per meeting).

**Table 2:** Results of logistic regressions of the probability of favourable and provisional opinions, treating each application as the unit of analysis

	Favourable opinion			Provisional opinion		
Variable	OR	95% CI	p Value	OR	95% CI	p Value
Year (2013 vs 2012)	0.73	0.56 to 0.96	0.025	1.32	1.02 to 1.71	0.034
EO vs comparator	0.80	0.35 to 1.84	0.59	1.10	0.50 to 2.42	0.82
Year×(EO vs comparator)	1.35	0.80 to 2.29	0.26	0.77	0.48 to 1.27	0.31
CTIMP status (no vs yes)	2.34	1.62 to 3.37	<0.001	0.38	0.26 to 0.54	<0.001
% interventional (per percentage point)	1.00	0.99 to 1.01	0.90	1.00	0.99 to 1.01	0.50
Workload (studies per meeting)	0.88	0.70 to 1.09	0.24	1.01	0.82 to 1.24	0.97

CTIMP, Clinical Trials of Investigational Medicinal Products; EO, ethics officer.

A diagnostic measure for both analyses showed that only 5% of the variation in opinion outcomes was explainable using the variables analysed. The statistical analyses thus fall far short of providing comprehensive explanations of RECs' decision-making.

### Time taken to issue an opinion

Clock times were available for all applications, except where final opinions had not been issued at the time data were retrieved (mid-December 2013). Times were unavailable for 5 applications to ethics officer RECs (1.3% of the total) and 27 applications to comparator RECs (2.5%). A minimal difference in clock times between ethics officer and comparator RECs was found. A modest decrease in clock times from 2012 to 2013 occurred in the ethics officer RECs (33.3 vs 32.0 days), while comparator RECs' clock times increased slightly (32.6 vs 32.9 days), but the effect was not statistically significant (p=0.22) (table 3).

**Table 3:** Time taken to issue an opinion (clock times)

Number of applications, mean (SD) days to final opinion	May–November 2012 (baseline)	May–November 2013 (intervention period)	Total
Ethics officer REC	33.3 (11.9), n=171	32.0 (9.6), n=182	32.6 (10.8), n=353
Comparator REC	32.6 (13.2), n=531	32.9 (10.7), n=502	32.7 (12.0), n=1033
Total	32.8 (12.9), n=702	32.6 (10.4), n=684	32.7 (11.7), n=1386

REC, research ethics committee.

Clock times showed no statistically significant effect of year, ethics officer versus comparator, nor the interaction between them. The interaction term, had it been significant, would have indicated a reduction in clock times in connection with the presence of ethics officers.

Clock times varied significantly by the REC's CTIMP status (32.9 vs 28.3 days, flagged to review CTIMPs vs not flagged), by the percentage of interventional studies reviewed, and by the REC's workload. Busier RECs (reviewing over five studies per meeting) took on average two days longer to issue a final opinion than less busy RECs (33.7 vs 31.7 days (*F* 18.13, p=<0.001) (table 4). As with the analysis of favourable and provisional opinion rates, the analysis of variance (ANOVA) explained only 5% of the variation in clock times between applications.

**Table 4:** Analysis of variance of clock times

Variable	*F* ratio	p Value
Year (2013 vs 2012)	0.33	0.57
EO vs comparator	0.39	0.53
Year×(EO vs comparator)	1.51	0.22
CTIMP status (no vs yes)	8.67	<0.001
% interventional (per percentage point)	20.75	<0.001
Workload (studies per meeting)	18.13	<0.001

CTIMP, Clinical Trials of Investigational Medicinal Products; EO, ethics officer.

### Applicant survey

Responses to the survey were received from 51/171 (30%) of the applicants in the ethics officer RECs. Though all ethics officers reported that they had circulated the questionnaire, no responses were received from applicants to two of the ethics officer RECs. Generally, respondents were satisfied with the ethics officer intervention; most agreed that the role (87%) and the advice given (81%) were useful (table 5). Open-ended responses suggested that applicants valued the support and reassurance provided by the ethics officer.

Helpful just to have a general conversation to explore certain issues and options—the formal meeting can all feel quite formal and potentially defensive rather than supportive—Applicant 13

**Table 5:** Applicants agreeing or strongly agreeing with survey statements

Survey statements	Number (%) ‘agreed’ or ‘strongly agreed’
I felt better prepared prior to attending the REC meeting	39/51 (76)
I felt more able to answer the questions from the REC	28/51 (55)
I needed to do further work following my conversation with the ethics officer	13/51 (25)
Any further work I did was a waste of time	4/51 (8)
The advice given by the ethics officer was useful	42/51 (82)
The advice given by the ethics officer was not useful	0/51 (0)
My involvement in the ethics officer pilot was tiresome	1/51 (2)
I'm glad I took part in the pilot	35/51 (69)
I believe the work conducted by the ethics officer was beneficial to me	39/51 (76)
If given the option again in the future, I would choose to take part in the pilot	39/51 (76)
In hindsight, I wish I had chosen to not participate in the pilot	0/51 (0)
I received a more favourable decision because of the help given by the ethics officer	14/51 (27)

REC, research ethics committee.

Potential improvements to the ethics officer function were also identified by applicants, including the need for better communication between the REC and the ethics officer and a more well-defined remit for the ethics officer. Twelve respondents suggested that the support should be available before the application was submitted for review.

The only other option I could imagine working would be the option to submit your documents to the Ethics Officer prior to ethics submission. This way you would be submitting a more robust application and time to a favourable ethical opinion would be reduced.—Applicant 7

### Documentary analysis

Ten applications were excluded from the documentary analysis as a draft version of the ethics officer review form that had only been intended for use during familiarisation, had been used. In one committee (REC4), only three review forms were completed for the 30 applications submitted during the intervention period, so all documents relating to this REC were excluded to ensure comparability between RECs. Across the remaining five RECs, 14 applicants opted out of the ethics officer pilot and their applications were not included in the documentary analysis.

Of the 171 applications to ethics officer RECs in the baseline period, 110 (64%) applications had full documentary data (opinion letters and minutes) (table 6). Of 192 applications to ethics officer RECs during the intervention period, 100 (52%) had full documentary data (opinion letters, minutes and review forms).

**Table 6:** Applications to ethics officer RECs with full documentary data available for process evaluation

	Proportion of favourable applications available	Proportion of provisional applications	Proportion of unfavourable applications	Proportion of all applications included
Baseline period				
REC1	10/11	19/19	0/0	29/29 (100%)
REC2	8/9	20/21	0/1	28/31 (90%)
REC3	0/17	5/7	0/5	5/29 (17%)
REC4	0/6	0/22	0/1	0/30 (0%)
REC5	8/9	11/14	5/5	24/28 (86%)
REC6	4/4	15/15	5/5	24/24 (100%)
Total for all RECs combined	30/56 (54%)	70/98 (71%)	10/17 (59%)	110/171 (64%)
Intervention period				
REC1	2/6	15/27	2/4	19/37 (51%)
REC2	6/8	12/?	1/?	19/25 (76%)
REC3	5/19	5/?	3/?	13/37 (35%)
REC4	0/18	0/10	0/1	0/29 (0%)
REC5	8/11	16/20	1/1	25/32 (78%)
REC6	5/6	15/19	4/6	24/31 (77%)
Total for all RECs combined	26/68 (38%)	63/110 (57%)	11/19 (58%)	100/192 (52%)

REC, research ethics committee.

For the ethics officer intervention to work, RECs needed to convert some opinions that would otherwise have been provisional opinions into favourable opinions, on grounds that the revisions that had been identified had been agreed with the applicants in advance. However, review of the ethics officer review forms suggested that the ethics officer and the principal applicant did not in fact discuss the application in advance of the REC meeting, as intended, in 31 of the 100 applications (for which data were available) in the intervention period. This happened for a variety of reasons: sometimes the attempt at contact did not happen or failed, or the applicants did not respond or were unavailable. The practical consequence was that in almost a third of cases the intervention did not function as intended. A further problem, implicated in four provisional opinions, was that the applicants did not address the advice given by the ethics officer in time. On at least one occasion, this was because the applicants were only notified of the ethics officer review on the day of the full REC review.

### Issues in applications and requests for action

The ethics officers' review form contained eight headings including a heading relating to ‘issues’ (see box 1). Review of completed forms found that not all forms contained entries under each heading; ethics officers sometimes left subsections blank.

Ethics officers identified issues in most (87/100) applications at pre-review, with a mean of 4.72 issues per application. The issues they most frequently identified were those relating to confidentiality, design and conduct of studies, and specific errors in participant information sheets and consent documentation (table 7). On average, ethics officers identified 3.2 issues in applications that went on to receive favourable opinions, compared with 4.9 in those that received provisional opinions and 6.9 in those that received unfavourable opinions, suggesting some degree of alignment between their review and the eventual opinion by the REC.

**Table 7:** Number and type of issues identified by ethics officers when they pre-reviewed applications. Source: ethics officer review forms

	Number of issues (number of applications affected*)			
	Favourable opinion (n=26)	Provisional opinion (n=63)	Unfavourable opinion (n=11)	All opinions (n=100)
Document issues				
Consent documents, including patient information sheets	7 (4)	34 (20)	3 (3)	44 (27)
Other documentation (eg, protocols, intervention descriptions)	21 (10)	93 (44)	26 (11)	140 (65)
REC application	12 (8)	32 (23)	7 (6)	51 (37)
Subtotal	40 (12)	159 (52)	36 (11)	235 (75)
Ethical issues				
Participant care	13 (6)	31 (24)	8 (5)	52 (35)
Confidentiality	11 (7)	46 (27)	8 (5)	65 (39)
Informed consent	3 (2)	16 (16)	5 (5)	24 (23)
Community issues	5 (3)	7 (7)	1 (1)	13 (12)
Subtotal	32 (13)	100 (43)	22 (8)	154 (64)
Study issues				
Design and conduct	8 (7)	33 (25)	13 (7)	54 (39)
Recruitment	4 (4)	20 (14)	5 (3)	29 (21)
Subtotal	12 (10)	53 (32)	18 (8)	83 (50)
Total number of issues (number of applications affected)	84 (18)	312 (58)	76 (11)	472 (87)
Mean issues per opinion category	3.2	5.0	6.9	4.7

*One application can generate more than one issue.

In four cases, the ethics officer correctly identified in advance that a provisional opinion was the likely outcome; regardless of any proposed amendments agreed with the applicants, they were of the view that the application would need to be re-reviewed by the REC. Ethics officers also identified two cases in which they did not foresee potentially preventable provisional opinions. In one of these, the ethics officer wrongly advised the researcher that it was not necessary to attend the REC meeting. In the other, the ethics officer did not anticipate issues that the REC saw as important.

For over a fifth (22%) of all applications, ethics officers did not identify in advance any issues likely to cause the applications to not receive a favourable opinion. Of these applications, fewer than half (10) received a favourable opinion. The remaining 12 applications received a provisional opinion. One contained an evident administrative error (incomplete application) that potentially could have been identified by the ethics officer.

As might be expected, RECs were typically much more detailed in their opinion letters than were ethics officers in their review forms, and accordingly the mean number of issues with applications recorded by RECs was higher than those recorded by ethics officers; rates of issue identification are therefore not comparable between RECs and ethics officer. Issues were raised by RECs for almost all applications (108/110 (98%) before the intervention and 96/100 (96%) during the intervention; table 8). There was little difference in the number of issues RECs identified per application during the baseline (12.5 issues per application) and intervention (11.4 issues per application) periods (table 8).

**Table 8:** Issues with applications identified by RECs before and during the ethics officer intervention. Source: REC meeting minutes and opinion letters

	Baseline (n=110 applications)			Intervention period (n=100 applications)		
Type of issue	Number of issues	Number (% of all) applications with an issue	Mean number of issues per application	Number of issues	Number (% of all) applications	Mean number of issues per application
Document issues						
Consent documents	45	27 (25)	0.4	46	32 (32)	0.5
Other documentation	298	85 (77)	2.7	268	85 (85)	2.7
REC application	121	57 (52)	1.1	81	43 (43)	0.8
Subtotal	464	93 (85)	4.2	395	88 (88)	3.9
Ethical issues						
Participant care	249	89 (81)	2.3	207	75 (75)	0.7
Confidentiality	147	68 (62)	1.3	132	57 (57)	0.6
Informed consent	30	23 (21)	0.3	31	24 (24)	0.2
Community issues	10	10 (9)	0.1	20	18 (18)	0.2
Subtotal	436	101 (92)	4.0	390	84 (84)	0.8
Study issues						
Design and conduct	299	93 (85)	2.7	225	78 (78)	0.8
Recruitment	179	82 (75)	1.6	131	65 (65)	0.6
Subtotal	478	104 (95)	4.3	356	88 (88)	0.9
Total issues	1378	108 (98)	12.5	1141	96 (96)	11.4

REC, research ethics committee.

Though RECs and ethics officers differed in the level of detail and the number of issues they raised, they could be compared in terms of the *types* of issues they identified. RECs and ethics officers identified the same types of issues in 75/100 (75%) applications. Thus, for example, the RECs and ethics officers identified an issue with the consent documentation in 17% of applications, and also were consistent in identifying no issues with consent in 58% applications. However, in 10 applications in which the ethics officer identified issues with consent documentation, the REC did not identify any. Conversely, the REC found fault with the consent form in 15 applications where the ethics officer did not.

Ethics officers recorded 296 issues that they documented as having discussed with the REC. For 250 of these, they recorded whether the REC agreed or disagreed with the specific point the ethics officer was making. The REC and ethics officers generally were in agreement in their views on ethical (72 out of 76 issues) and study issues (40 out of 42 issues) and on issues relating to participant documentation (123 of 132).

The number of requests RECs made of applicants for revisions before and during the intervention period showed little change. Most applicants—97/110 (88%) in the baseline and 92/100 (92%) during the intervention—were asked to make some revisions (table 9) and the mean number of requests per application—6.4 before and 6.7 during—were similar. RECs sometimes gave provisional opinions even when it appeared that the revisions required were minor and easily met the criteria for a favourable with conditions opinion (eg, only documentation issues were involved).

The patient information sheet needs rewriting in a less Americanised format […] the standard NRES regulatory clause should be included in the consent form.—REC6 (provisional opinion letter)

**Table 9:** RECs' requests for revisions by applicants before and during the intervention. Source: REC meeting minutes and opinion letters

	Baseline (n=110 applications)		Intervention (n=100 applications)	
	Number of revisions requested	Number (% of all) applications	Number of revisions requested	Number (% of all) applications
Documentation revisions				
Consent documentation	84	44 (40)	74	46 (46)
Other documentation	373	89 (81)	310	86 (86)
Subtotal	457	93 (85)	384	89 (89)
Procedural revisions				
Consent procedures	8	7 (6)	17	17 (17)
Data storage procedures	1	1 (1)	0	0 (0)
Recruitment procedures	7	6 (5)	0	0 (0)
Study design	6	6 (5)	7	7 (7)
Study protocol	15	12 (11)	14	12 (12)
Subtotal	37	24 (22)	38	29 (29)
Further information to be submitted				
Study design	69	38 (35)	58	28 (28)
Further documentation	19	17 (15)	9	8 (8)
Ethical practice	71	50 (45)	63	33 (33)
Improved application	7	6 (5)	13	9 (9)
Protocol and procedures	1	1 (1)	47	33 (33)
Researcher credentials	33	27 (25)	7	7 (7)
Other	12	12 (11)	0	0 (0)
Subtotal	212	77 (70)	197	58 (58)
Total	706	97 (88)	619	92 (92)

REC, research ethics committee.

## Discussion

This controlled study found that an ethics officer intervention did not increase the proportion of applications to RECs that were approved first time, nor did it reduce the time to a final opinion. Though the process evaluation suggested that applicants generally valued the ethics officer service, that ethics officers were successful in identifying many issues with applications, and that RECs and ethics officers demonstrated good alignment in their views on whether issues were justified, it also provided some insights into the reasons for the null result. A major challenge was that the intervention did not function exactly as designed. For instance, officers did not reach applicants in advance of meetings in almost a third of applications for which data were available, and they did not consistently anticipate issues in applications that went on to get provisional opinions. Perhaps more crucially, however, the behaviour of RECs did not appear to change in response to the ethics officer intervention. They did not appear to take reassurance from the ethics officer process that they would not need to re-review changes to applications. These findings are disappointing, in that they indicate that an ethics officer role as implemented does not provide a straightforward fix for some of the current challenges in obtaining timely ethical approval for studies. They also suggest potential targets for improvement.

It is clear that a large number of avoidable errors continue to be made by applicants at the submission stage. Reducing these might help in improving the rate of favourable opinion at first review, not least by reassuring RECs that applicants have engaged seriously with the process. It is possible that improvements to the application process itself (eg, better clarity, more user-friendly instructions) might help in reducing such errors, but also likely to be important is more attention by applicants themselves to the details of applications, including consistency between different parts of applications, grammatical correctness and compliance with application instructions. Better training and education of applicants may well be the key to this, and here organisations in which applicants are based might take on a more comprehensive role than they do at present. More thorough checking of applications before submission, for example by project sponsors, might be helpful, since sponsors have a formal role in oversight of research. It is unclear whether having ethics officers act as proof-readers would be appropriate, though they might be able to advise applicants at an early stage if an application does not appear to reach the required standard. Also clear is that some applications involve more complex ethical or procedural issues, where earlier sensitisation by applicants to issues likely to be of concern to RECs might be helpful. Here, upfront discussion with an ethics officer might be of value before an application is submitted.

Better preparation by applicants might go some way towards reducing delays and improving the experiences of applicants at research ethics review. However, a more comprehensive solution is likely to be elusive without changes in the norms and behaviours of RECs themselves. The evidence in this study and others^24–26^ suggests that RECs find it difficult to resist identifying issues with applications (virtually none escaped without an issue either before or during the intervention), and that they tend to default to provisional opinions even when an approval with conditions would be a reasonable option. Previous analyses have suggested that this may be because RECs see the production of a list of issues as a display of their own diligence and as serving the interests of accountability.^27^ ^28^ Appropriately targeted educational interventions may be helpful in addressing these problems, as may reinforcement of new norms through accountability mechanisms—for example, RECs might be asked to provide additional explanation or rationale for a random sample of decisions each year, perhaps through a reciprocal peer review arrangement with other RECs. Qualitative work may be especially useful in understanding the impact of such arrangements on decision-making.^9^ ^10^

This study has a number of limitations. RECs had volunteered for the task, and knew that they were being studied; this may have influenced their behaviour and decision-making. The ethics officers themselves, as experienced chairs of RECs, may not have been fully representative of those likely to occupy such roles in real life, nor is it clear that the ethics officers fully or consistently complied with the instructions they were given—raising questions about intervention fidelity. It is possible that applications for which data were not available were different in some way. The statistical analyses explained only small percentages of the variation in outcomes, possibly because variables important for the context were not available. Clock times were missing for a small number of applications. It was not possible to choose comparator RECs that matched the ethics officer RECs entirely as had been planned. Interviews with applicants, ethics officers and REC members, chairs and staff would have been useful, as would ethnography of REC meetings, but were not possible for budgetary reasons. The process evaluation was challenged by missing data: only just over half of applications in the intervention period, and just under two-thirds in the preintervention period, had full documentary data available.

## Conclusions

Addressing challenges in the governance of research is unlikely to be straightforward. This controlled study suggests that an ethics officer intervention, at least as designed and implemented in this project, does not confer advantages in terms of increasing rates of approvals at first review of applications by a research ethics committee nor in decreasing time to committee decision. Improvements are likely to require changes in the culture and behaviours of applicants and research ethics committees. Other forms of the ethics officer role—for example one that provides more upfront support prior to formal submission—might also be valuable and would benefit from evaluation. Future studies of this kind of intervention might usefully standardise the training, eligibility thresholds and precise roles of those serving as ethics officers, and, subject to systems amendments being made to enable it, allow the ethics officers and consenting applicants to amend their applications before formal ethics review.
